# Medical illustrations in radiologic reports for endometriosis: improving communication with surgeons and patients

**DOI:** 10.1186/s13244-026-02307-3

**Published:** 2026-05-22

**Authors:** Marcela Caetano Vilela Lauar, Brunna Clemente Oliveira, Karina de Souza Giassi, Izabela Pires Franco, Ana Luísa Alencar De Nicola, Alice Brandão, Isabelle Thomassin-Naggara, Duarte Miguel Ribeiro, Gladis Maria Pacileo Anchieta Rodrigues Ribeiro, Luciana Pardini Chamié

**Affiliations:** 1 Chamié Imagem da Mulher, São Paulo, Brazil; 2 Hospital Israelita Albert Einstein, São Paulo, Brazil; 3 LUCE Radiologia Contemporânea, Bento Gonçalves, Brazil; 4 Clínica Izabela Pires Franco, Belém, Brazil; 5 Faculdade de Ciências Médicas da Santa Casa de São Paulo, São Paulo, Brazil; 6 Fleury Medicina e Saúde, São Paulo, Brazil; 7 Fonte Imagem, Rio de Janeiro, Brazil; 8 Sorbonne Université, Paris, France; 9 Clínica Duarte Miguel Ribeiro, São Paulo, Brazil; 10 Brigham and Women’s Hospital, Boston, MA USA

**Keywords:** Diagnostic imaging, Endometriosis, Medical illustration, Radiology, Communication

## Abstract

**Abstract:**

Endometriosis is a complex, multicompartmental disease in which accurately conveying the extent of involvement to the surgical team is essential for preoperative planning and patient counseling. In this context, medical illustrations have gained increasing popularity as a valuable complement to traditional radiology reports. These drawings provide a visual summary of imaging findings, which are often lengthy and challenging to interpret in written form alone. By translating complex imaging data into accessible visual representations, illustrations can enhance communication between radiologists and surgeons, ultimately leading to improved surgical outcomes. Surgeons may also use these illustrations during patient consultations to explain the disease’s involvement and the planned surgical approach. They can also be applied to patients conservatively managed to monitor treatment efficacy and clinical evolution. This not only improves patient understanding but also saves valuable time during appointments—time that can instead be dedicated to addressing patient concerns and strengthening the physician–patient relationship. Importantly, in modern, patient-centered care models, individuals are encouraged to take an active role in treatment decisions. By helping patients visualize and better understand their condition, medical illustrations empower them to engage more meaningfully in shared decision-making processes. These visual tools enhance treatment adherence and facilitate a more compassionate and efficient clinical workflow.

**Clinical relevance statement:**

Endometriosis imaging is complex to communicate due to the multifocal nature of the disease and anatomic distortion. Drawings can complement reports, thereby improving surgical planning and patients’ understanding while also enhancing radiologist engagement.

**Key Points:**

- Endometriosis imaging reporting can be complex, and illustrations can help convey its extent in imaging for effective surgical planning.
- Medical illustrations also offer multiple benefits for patients, surgeons and radiologists.
- Multiple tools are currently available to assist with creating drawings, eliminating the need for specific artistic skills.

**Graphical Abstract:**

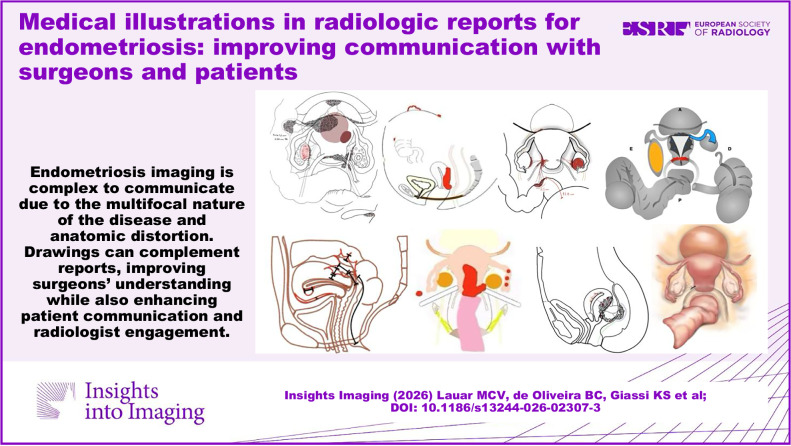

## Introduction

Endometriosis is a common gynecologic condition for which surgical management is frequently an appropriate treatment option [1–3]. Achieving successful surgical outcomes (a single, complete and definitive procedure without complications) relies heavily on accurate preoperative disease mapping [4, 5].

Precise identification of lesion location and organ involvement enables appropriate surgical planning, including selection of the surgical team, required instrumentation, estimated operating room time and adequate patient counseling, including possible complications. Because preoperative staging is based on clinical evaluation combined with dedicated imaging, such as ultrasound (US) with bowel preparation and magnetic resonance imaging (MRI), radiologists play a central role in this process [1–3, 6]. However, the multifocal nature of endometriosis and its tendency to distort normal pelvic anatomy often make image interpretation and clear multidisciplinary communication of findings challenging in daily practice [7].

**Fig. 1:**
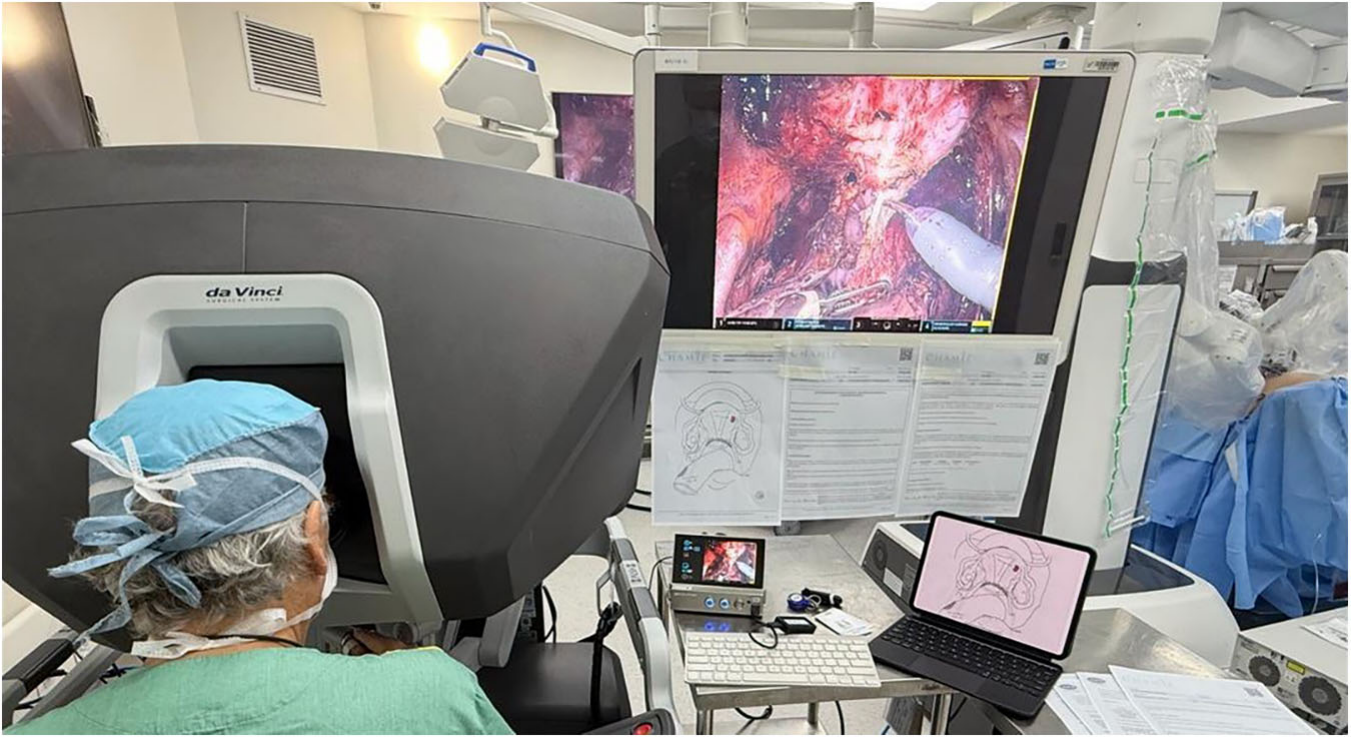
Photograph of a surgeon performing laparoscopic robotic surgery for endometriosis, with the imaging report and corresponding illustration placed nearby the console to guide the procedure

Medical illustrations depicting endometriotic involvement have emerged as a valuable tool for conveying imaging findings [2, 8] (Fig. 1), as they provide a unique representation of the disease distribution for each individual. Used to complement traditional reports, they serve as a visual fingerprint, synthesizing complex imaging information in an intuitive visual format. This visual approach may benefit surgeons, patients, and radiologists in different but interconnected ways, as explored throughout this article.

This article aims to highlight the advantages of incorporating drawings as a complement to endometriosis imaging reports and provides a practical guide for radiologists interested in starting and refining this practice.

## Interdisciplinary communication: supporting surgical teams

A clear understanding of findings supports surgical planning, allows more accurate estimation of operative complexity and duration, and contributes to optimized hospital resource utilization—an important consideration for women with endometriosis who are often of working age. It also plays a key role in comprehensive preoperative patient counseling [2, 5, 9, 10]. Therefore, efforts have been made to improve multidisciplinary communication by addressing how radiologists describe imaging findings [11], especially in complex conditions such as endometriosis.

Structured and compartmentalized reporting has become increasingly relevant in this setting to ensure consistent anatomical assessment. A study by Barbisan et al showed that the use of structured MRI reports for pelvic endometriosis outperformed traditional reports in fully documenting essential disease features and may reduce the gap between experts and less-experienced radiologists [5], and is currently recommended by both the European Society of Urogenital Radiology (ESUR) [8] and the Society of Abdominal Radiology (SAR) consensus statements on endometriosis imaging [12].

**Fig. 2:**
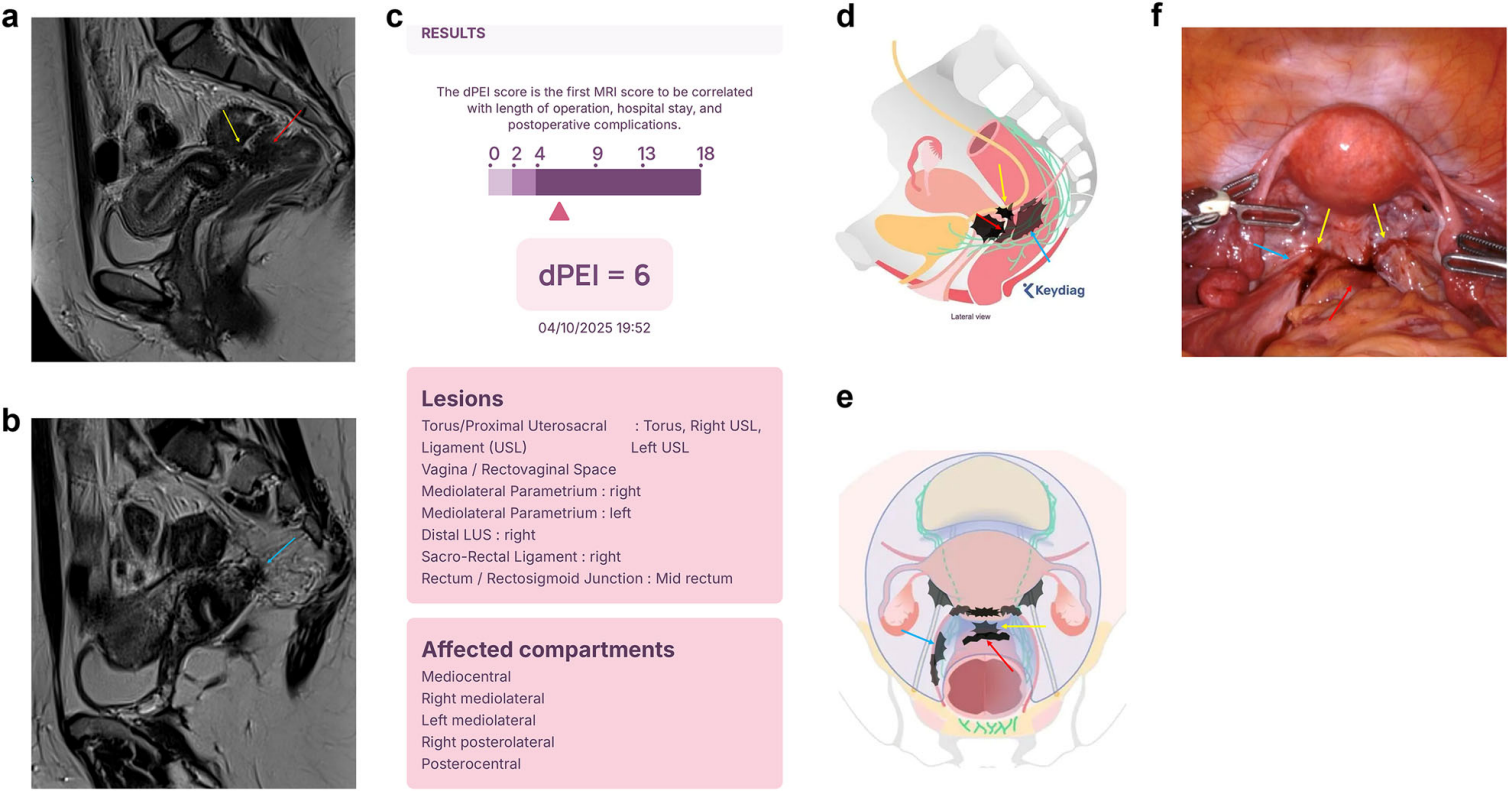
**a**–**f** A 37-year-old nulliparous patient on day 7 of the menstrual cycle was referred for endometriosis mapping in the context of infertility (in vitro fertilization failure). She also reported dysmenorrhea, dyspareunia, and dyschezia. Sagittal T2-weighted MRI images show deep endometriosis involving the retrocervical (yellow arrow) and paracervical (blue arrow) regions with bowel involvement (red arrow) and posterior cul-de-sac obliteration (**a**, **b**). The dPEI calculator was used to estimate surgical risk (**c**), and illustrative drawings were automatically generated by the app to represent the disease (**d**, **e**). The patient subsequently underwent videolaparoscopy (**f**), and the findings were consistent with the MRI results (in **a**, **b**, **d**–**f**, the yellow arrow represents the retrocervical lesion, blue arrow represents the paracervical lesion, and red arrow represents the bowel lesion)

Classification systems to help predict surgical difficulty have also been developed. An ideal classification should be used for both imaging specialists and surgeons. To date, there are two imaging-based tools proposed for preoperative evaluation of surgical complexity: the #Enzian classification [13], applicable to both ultrasound and MRI reporting [14], and the Deep Pelvic Endometriosis Index (dPEI) MRI-based scoring system developed by Thomassin-Naggara et al [1]. The dPEI calculator predicts surgical complexity based on imaging findings (Fig. 2) and is publicly available (https://www.dpei-score.org).

There is also a study by Abrão et al that used US findings in predicting the laparoscopy complexity defined as the 2021 American Association of Gynecologic Laparoscopists (AAGL) Endometriosis Staging, which also offers an automated calculator capable of generating both the score and a corresponding illustrative drawing [15].

**Fig. 3:**
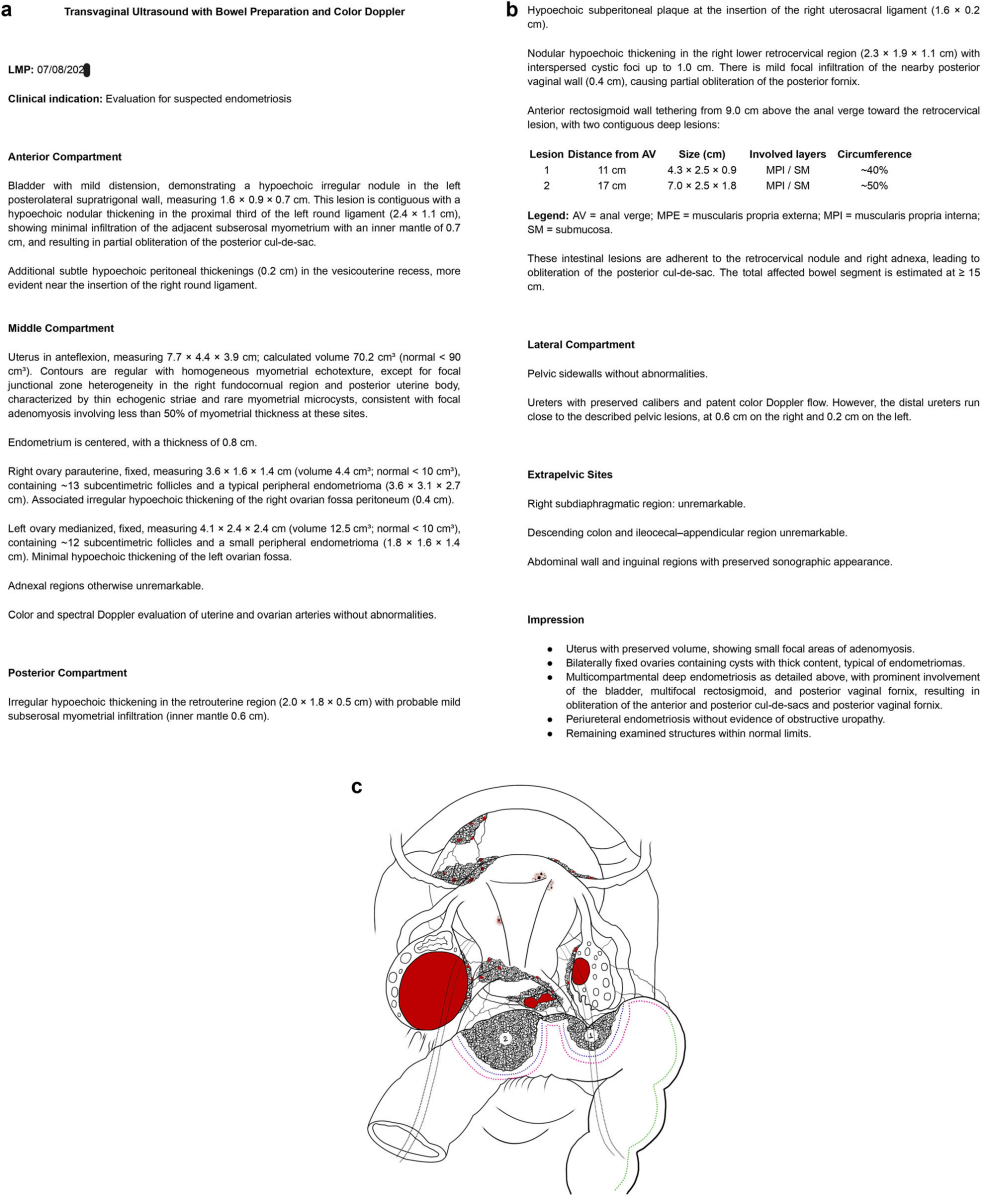
A comprehensive two-page transvaginal ultrasound report with bowel preparation and color Doppler for endometriosis mapping is shown (**a**, **b**). **c** presents all the described findings in a more intuitive single diagram

**Fig. 4:**
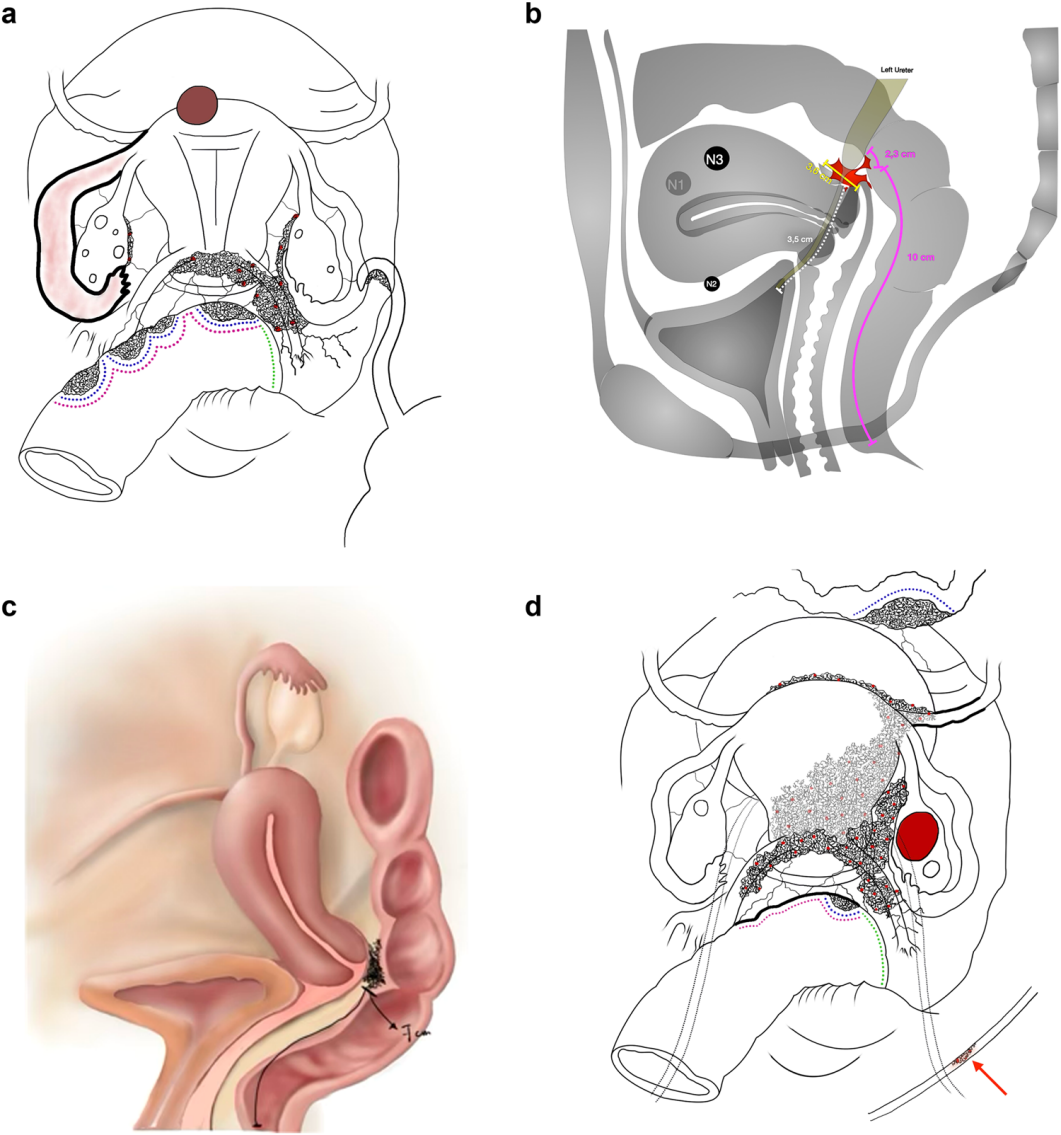
**a**–**d** Illustrations from different patients, created by different radiologists, summarizing key information for preoperative planning in multiple compartments, as well as other associated conditions that may coexist in the female pelvis. Illustration demonstrating deep endometriosis (DE) with left hematosalpinx, an appendiceal nodule and multifocal intestinal lesions. Relevant surgical details include the extension of each bowel lesion (blue dotted lines), the estimated total length of the affected bowel segment (pink dotted line) and the inferior distance from the anal verge (green dotted line) (**a**). Illustration showing DE in the retrocervical region, with rectosigmoid infiltration, depicting lesion extent and the distance from the anal verge. It also demonstrates left ureteral involvement, with upstream dilatation and indicates the distance (in centimeters) from the ureterovesical junction to the site of the obstruction (white dotted line) (**b**). Illustration of deep endometriosis in the posterior vaginal fornix involving the adjacent bowel, with measurement of the distance from the anal verge (**c**). Illustration of extensive multifocal deep endometriosis, with posterior myometrial infiltration, involvement of the ileum and rectosigmoid, and a diaphragmatic endometriotic lesion (red arrow). There is also a right ovarian endometrioma and a low antral follicle count (**d**)

Similar to structured reports and preoperative classifications, visual representations can be used as an effective tool to organize and communicate imaging findings in a 3D perspective. Several information that may impact the surgical strategy can be effectively conveyed through drawings (Fig. 3). For instance, in cases of bowel involvement, factors such as the distance from the anal verge, the degree of circumferential involvement, the number of bowel lesions and the longitudinal extent of the lesion influence the decision between shaving, discoid excision, or segmental resection [3, 16] (Fig. 4a). In the anterior compartment, measurements such as the distance between ureteral and bladder lesions and the ureterovesical junction, infiltration of the bladder wall, as well as the presence or absence of ureteric obstruction, can guide the choice between end-to-end anastomosis and ureteric reimplantation [3] (Fig. 4b). Lesions in the posterior vaginal fornix also require detailed information including extent and distance from the anal verge, considering lower lesions may require a deeper, subperitoneal dissection for complete resection (Fig. 4c) [17].

The importance of clearly communicating the involvement of non-gynecologic structures by endometriosis—such as the bladder, ureters, bowel, nerves, and diaphragm (Fig. 4d)—also relies on the fact that they require surgical planning that involves coordination with urologic, colorectal, neuropelvic, or thoracic surgeons, as well as the use of specific surgical tools and materials [1, 2, 18]. To enhance the understanding and practical application of an illustrative map, it is essential to align it with the perspective used in surgical procedures. This alignment is crucial because the surgical view is mirrored compared to that of a radiologist. Specifically, what appears on the surgeon’s right side will correspond to the radiologist’s left. By considering this difference in perspectives, users can effectively interpret and utilize the map in a medical context.

**Fig. 5:**
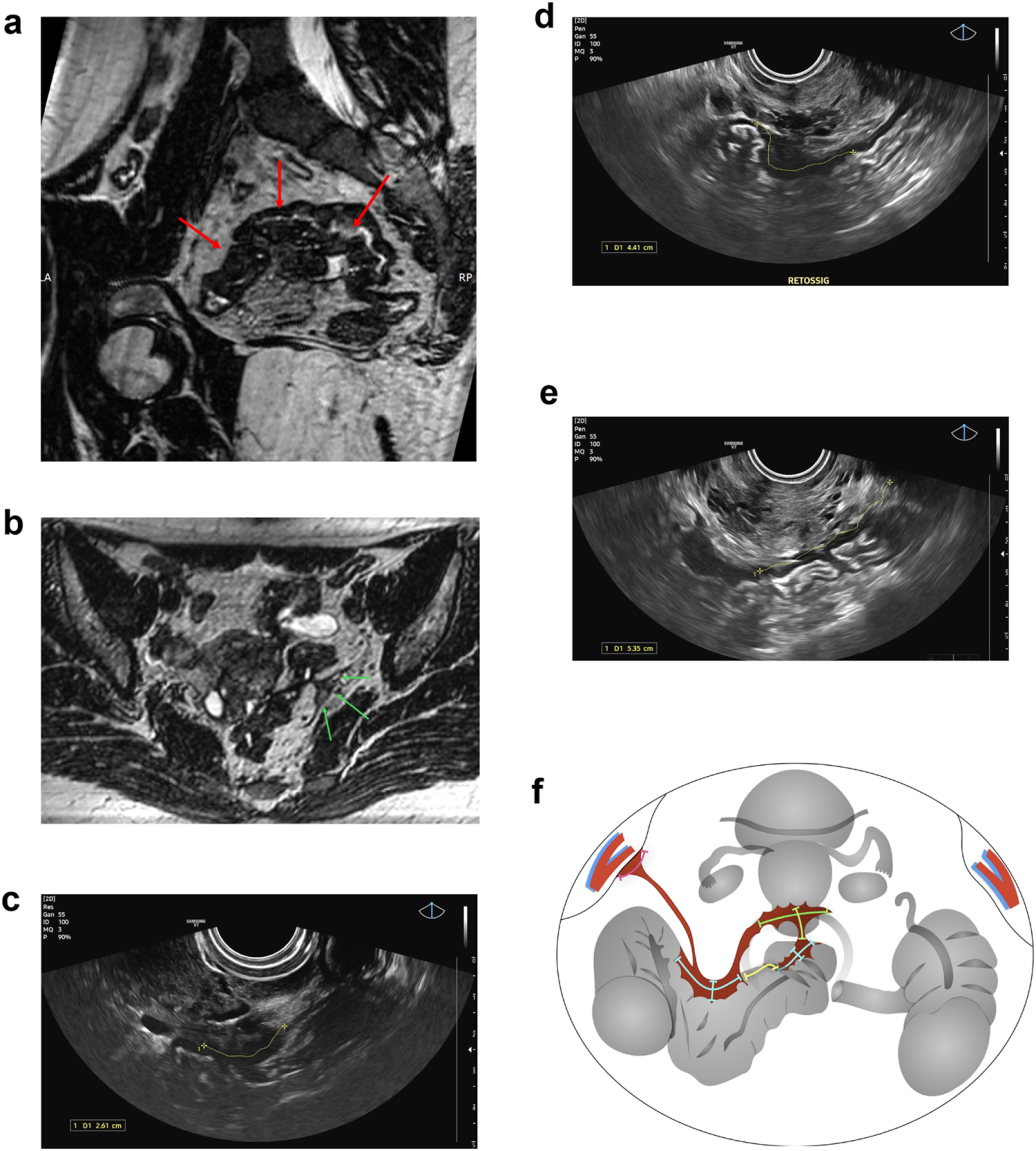
**a**–**f** A 23-year-old patient referred for endometriosis mapping. A 3D reconstruction of the volumetric MRI T2-weighted sequence suggested a single contiguous lesion (arrows in **a**). MRI also revealed lesion extension to the left pelvic sidewall (**b**). On ultrasound, bowel lesions were identified (**c**, **d**); however, they were better delineated as two distinct lesions, separated by an area of normal tissue (yellow line in **e**). The illustration created for the case synthesizes the findings from both modalities: extension to the left lateral pelvic wall (visible only on MRI) and the presence of two separate bowel lesions (more clearly characterized by ultrasound) (**f**)

Besides, a single, well-designed drawing can often condense pages of descriptive reporting into a clear and intuitive visual summary, sometimes even integrating findings from both TVUS and MRI in a unified report. Although these imaging modalities differ, they are complementary, and illustrations help unify this information, offering the surgeon a cohesive understanding of the overall disease burden (Fig. 5a–f).

**Fig. 6:**
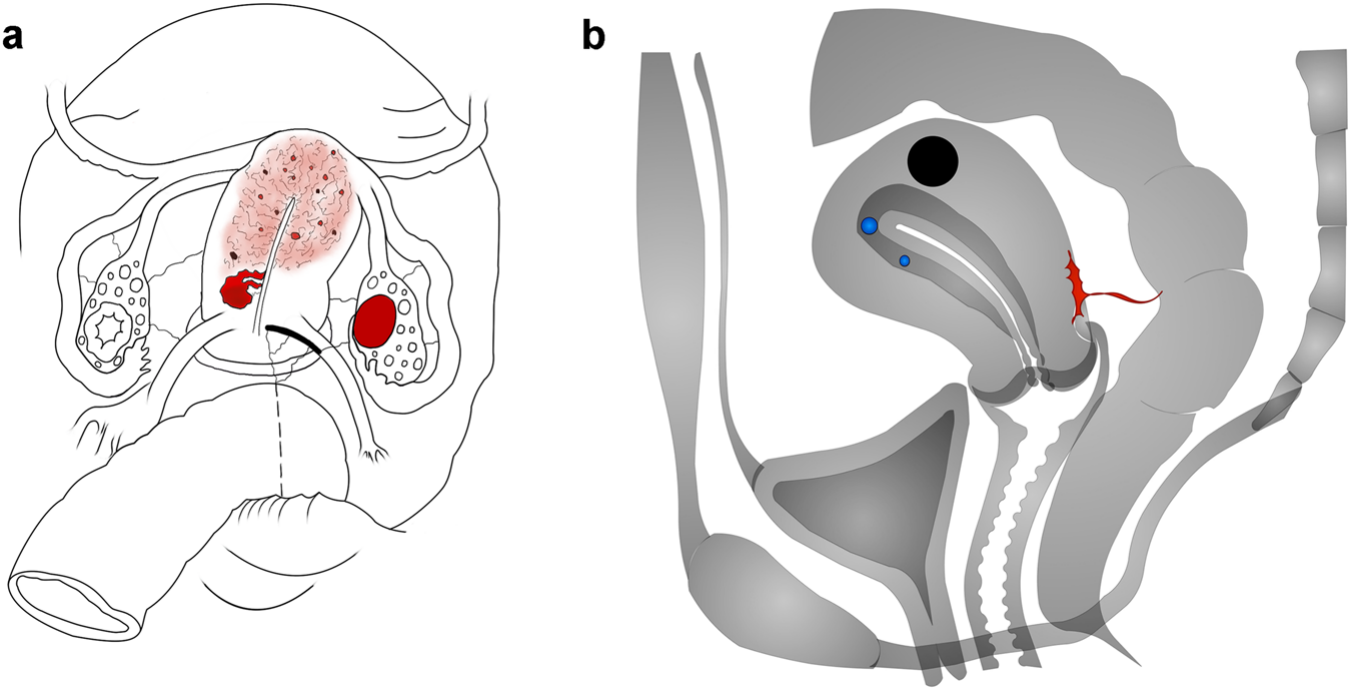
In this postoperative follow-up of endometriosis, the radiologist illustrated a right unicornuate uterus with adenomyosis, a right ovarian endometrioma, fibrotic changes in the right uterosacral ligament, and a rectosigmoidectomy anastomosis site (**a**). In this case, the color red was used to represent endometriosis in the retrocervical region, black circles indicate leiomyomas, and blue spheres illustrate cysts in the uterine junctional zone (adenomyosis) (**b**)

**Fig. 7:**
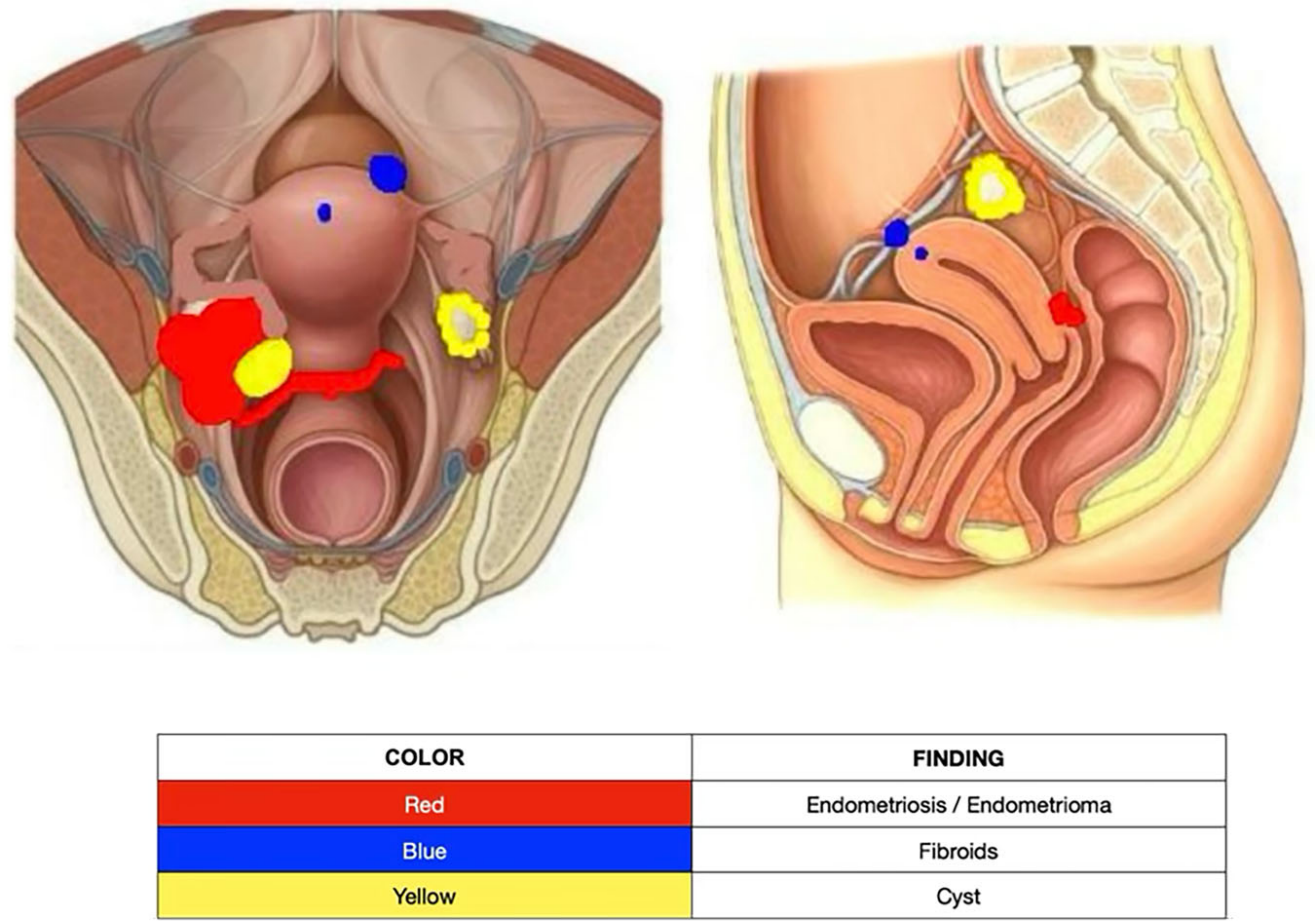
In this drawing, the radiologist illustrated foci of endometriosis, leiomyomas, and adnexal cysts in both the axial and sagittal planes, and included a table with a legend explaining the color coding, to further enhance the surgeon’s understanding

Importantly, although these imaging studies are typically requested to map endometriosis, other concomitant findings relevant to the clinical management of the disease—such as leiomyomas, adenomyosis, adhesions, and ovarian findings including the antral follicle count—can also be depicted in the illustrations. Many of these findings are common and may coexist with endometriosis. Incorporating them into the drawings allows for a more comprehensive and individualized depiction of the patient’s female pelvis, encompassing both endometriotic lesions and associated conditions (Fig. 6a, b). Over time, both radiologists and referring physicians can become familiar with these visual conventions, which may enhance long-term communication by allowing for quicker interpretation and shared understanding of findings (Fig. 7).

## Patient communication

In the setting of patient-centered care, patients are increasingly engaged in shared decision-making, shifting away from a traditionally passive role in treatment decisions dictated solely by physicians [8]. For this collaborative approach to be effective, patients must understand both their condition and available therapeutic options. Notably, patients are more likely to adhere to recommended treatments when they understand their disease [19]. However, the lack of access to clear, understandable information is one of the most frequently reported sources of dissatisfaction among patients with endometriosis [8].

Drawings offer practical advantages during consultations by helping individuals better understand their condition and the proposed management strategies. Visual information enhances comprehension and acceptance, promotes patient engagement, and facilitates shared decision-making. Many surgeons use hand-drawn sketches during consultations to convey the nature and extent of the disease [20]. Incorporating standardized illustrations into radiology reports can reduce consultation time otherwise spent creating explanatory drawings, allowing surgeons to focus on addressing patient concerns, providing emotional support, and facilitating information processing. It also empowers minimally invasive gynecologic surgeons (MIGS) and reproductive surgeons to provide clear and effective preoperative counseling regarding surgical techniques, associated risks, and realistic expectations, ultimately strengthening the patient-physician relationship.

Furthermore, radiologists themselves can act as a bridge between patients and surgeons. Although radiologists are often perceived as distant from direct patient care, studies have shown that direct communication between patients and radiologists after an initial image review can significantly improve the quality, clarity, and structure of radiology reports [21]. In this context, illustrations may provide a simpler, more appropriate vocabulary, thereby supporting patient understanding and enabling them to revisit and reflect on the information after the consultation. Overall, they can play a meaningful role in alleviating the burden on those living with endometriosis, which is especially relevant given the central role of imaging in endometriosis management. Radiology services have a significant impact on the patient’s clinical journey, and satisfaction with this process is closely linked to psychological well-being and treatment outcomes [22].

Moreover, when patients perceive that their physician is making a genuine effort to help them understand their condition, such as by using visual representations, this alone can strengthen the physician–patient relationship. This is particularly vital in endometriosis care, where many patients have experienced significant psychological distress after years of having their symptoms overlooked or normalized by healthcare providers [22]. Such experiences can erode trust in the healthcare system, underscoring our responsibility to help rebuild it [19].

In the context of endometriosis, pain invalidation and infertility represent deep emotional wounds for many women [22], and a visual representation of their condition can help validate their experiences by providing a tangible explanation for their symptoms. Being able to ‘put a face’ to a disease they cannot see, only feel, is deeply meaningful, as it transforms an invisible condition into something concrete and understandable. This can also empower patients to share their drawings with others, facilitating understanding and empathy within their personal and professional networks.

Nonetheless, medical illustrations can also evoke psychological discomfort, as they expose the inner workings of a disease that has often been a source of profound emotional and physical pain. Accordingly, the creation and even the educational sharing of such drawings must be approached with care and sensitivity, always respecting the patient’s emotional boundaries and privacy.

## Impacts for radiologists

For radiologists, the ability to express imaging findings fosters a more accessible and intuitive common visual language with the referring physician. When drawings address the specific informational needs of referring physicians, they not only enhance the clarity and reliability of the reports but also strengthen the radiologist’s professional credibility. Over time, this practice promotes a collaborative spirit and builds mutual trust across clinical teams. Furthermore, the illustrative map may be seen by the patient as a worthwhile investment in her overall quality of care, enhancing the radiologist’s fundamental place in surgical planning.

Actively engaging in visual art has also been shown to stimulate creative thinking, provide new perspectives on clinical scenarios, and strengthen neural connectivity—benefits that may counteract age-related cognitive decline and enhance overall engagement [23, 24].

In parallel, the healthcare community continues to face increasing levels of psychosocial stress and professional burnout, driven by high workloads, emotional strain, and systemic pressures [25]. Creative activities, including visual arts production, have demonstrated potential in mitigating burnout and improving overall well-being among healthcare professionals [24].

However, despite these advantages (Table 1), incorporating drawings into radiology reports can present some challenges. Radiologists may perceive it as an additional task in an already time-constrained workflow, particularly in high-volume practices. There may also be concerns about illustrating uncertain findings, as translating complex or ambiguous imaging data into a visual format may raise anxiety about potential misinterpretation or overcommitment to a diagnosis. Nevertheless, over time, especially in follow-up studies, previously created illustrations can serve as a clear and immediate reference, allowing radiologists to more easily understand what was previously reported than by relying solely on written text.

**Table 1:** Summary of the main advantages of drawings

Stakeholder	Benefits
Surgeons	• Faster understanding of disease extent • Surgical planning • Multidisciplinary coordination • Tailored informed consent
Patients	• Improved understanding • Shared decision-making • Validation of symptoms
Radiologists	• Improved communication and trust with referring physicians • Workflow efficiency in follow-up • Professional satisfaction

Recent developments in digital tools have begun to address these barriers. Some platforms now allow for semi-automated generation of illustrations based on structured report data or classification input, such as disease location and depth, reducing the manual workload and lowering the threshold for adoption. These tools offer a promising avenue for integrating visual communication into radiologic practice in a time-efficient and standardized manner [18]. Furthermore, in the future, the illustrative map added to a more direct structured report can become a less time-consuming endometriosis report.

## Methods of illustration: from traditional to immersive technologies

**Fig. 8:**
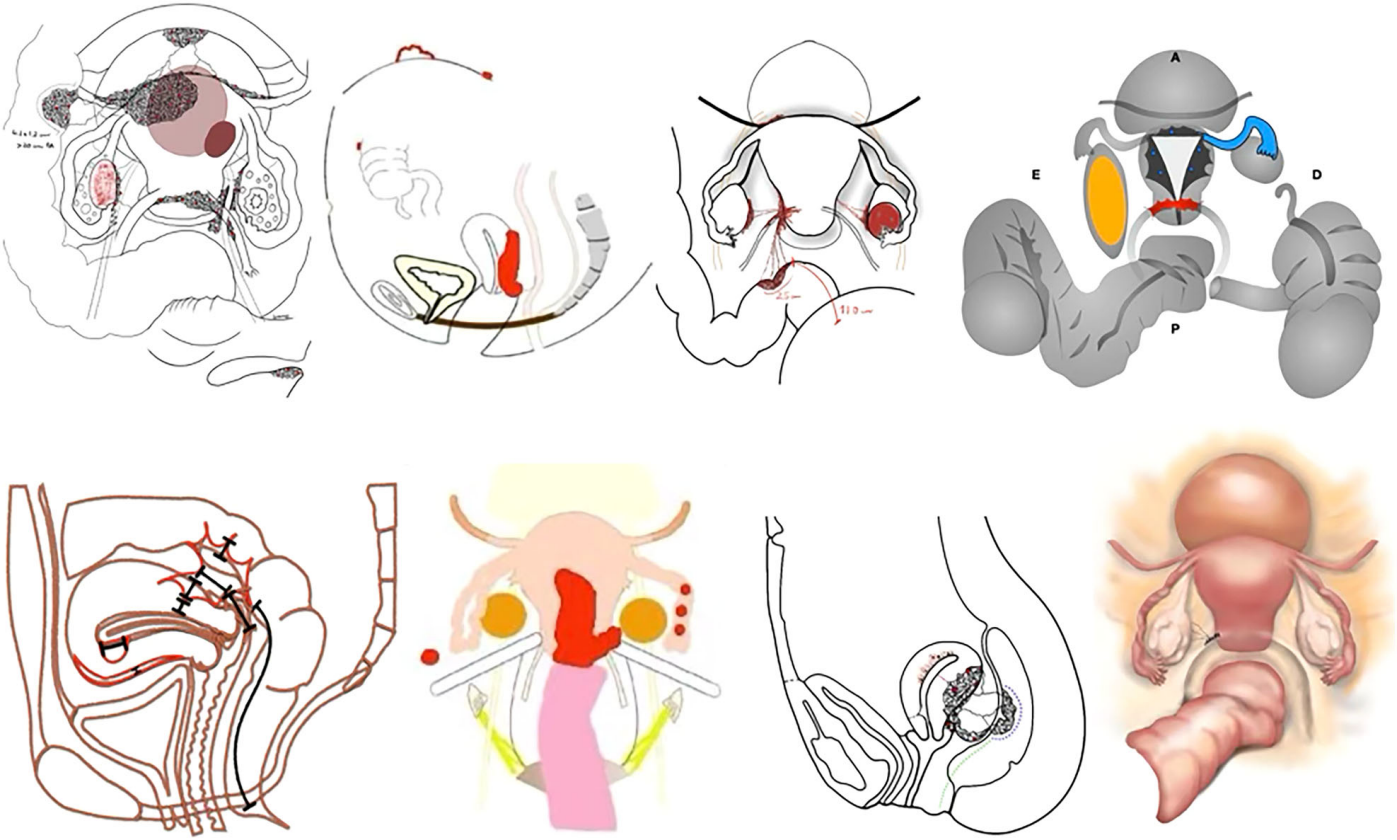
Endometriosis in different patients, illustrated by different radiologists using various methods, including pen-on-paper drawing, tablet, and computer-based software

While some healthcare professionals may perceive a lack of artistic training as a barrier to incorporating visual tools into clinical practice, evidence suggests that producing effective medical illustrations is entirely feasible without formal artistic expertise [26]. A range of tools is available to support the creation of medical illustrations, from traditional hand-drawn sketches to professional-grade digital platforms (Fig. 8). Among currently available tools, the Deep Pelvic Endometriosis Index (dPEI) application (previously introduced in this article) is freely accessible on computers and mobile devices, provides automated illustrative outputs based on MRI findings, and has been validated in European consensus recommendations for endometriosis imaging and reporting [8].

Hand-drawn tools are perfect when you need artistic flexibility to replicate subtle findings, like endometriomas or adenomyosis contours. Vector-based tools are ideal for schematic, labeled, and scalable illustrations, and they can be included directly in the report. Radiologists widely use Presentation Software due to its accessibility and ease of use, although it is less refined for producing professional illustrations. Table 2 presents a list of valuable tools for radiologists, categorized by their type of functionality.

**Table 2:** Digital tools for radiologists

Tool	Main use
Hand-drawing-based tools	
Procreate	High-precision freehand digital drawing; ideal for anatomical sketches.
Adobe Fresco	Combines raster and vector brushes; great for expressive clinical drawing.
Sketchbook (Autodesk)	User-friendly drawing app with a wide brush variety for quick illustrations.
Krita	Open-source tool with robust features for detailed medical illustrations.
Tayasui Sketches	Minimalist interface, good for fast sketching on iPads.
GoodNotes/Notability	Not primarily for illustration, but useful for marking radiology images.
Vector-based tools	
Concepts	Precision sketching with vector-based editing; ideal for tablets.
Adobe Illustrator	Industry standard for scalable vector graphics and anatomical diagramming.
Inkscape	Free alternative to Illustrator; useful for precise labeling and shapes.
Affinity Designer	Powerful, budget-friendly vector tool with raster-layer integration.
CorelDRAW	Professional-grade illustration software with extensive vector tools.
Figma	Collaborative UI/UX tool is also suitable for anatomical flowcharts.
Presentation software tools	
PowerPoint	Commonly used in radiology presentations, simple shapes and arrows.
Google Slides	Easy-to-use for collaborative annotation and basic diagramming.
Keynote	Apple-based; elegant templates and smooth annotation tools.
Canva	Intuitive templates; great for clean diagrams with minimal effort.

More recently, artificial intelligence (AI)-assisted tools have expanded the possibilities for generating customized medical visuals. Platforms such as BioRender (BioRender Inc.), Midjourney (Midjourney Inc., San Francisco, CA), and DALL·E (OpenAI) enable the creation of illustrations from text-based prompts, providing accessible solutions for users without formal training in graphic design. These tools reduce the barrier to visual content creation while maintaining an emphasis on anatomical clarity and scientific accuracy.

Importantly, the effectiveness of an illustration lies not in its artistic complexity but in its ability to convey key information. Simplified, schematic diagrams often enhance communication and comprehension more effectively than highly detailed anatomical drawings, particularly in the context of surgical planning and patient education [26]. Because these drawings are primarily intended to support surgeons, they should follow the surgical perspective: findings located on the patient’s left side should be represented on the left side of the illustration, and the same applies to the right side, in contrast to conventional radiological orientation.

There are now technologies available that enable the 3D mapping of endometriosis [7], including online programs. These tools offer advantages over 2D imaging technologies, such as the ability to visualize the disease from multiple angles and to render structures transparent, among other features.

To facilitate the adoption of illustrations in endometriosis reports, Table 3 provides a step-by-step guide on how to get started.

**Table 3:** A step-by-step guide on how to start drawing imaging findings

1	**Don’t be afraid to start**. Artistic skill is not a prerequisite—clarity of communication is paramount. Excessive self-criticism regarding artistic quality should not discourage the use of drawing as a clinical tool.
2	**Create a standard pelvic anatomy template and find a style that suits you**. Begin by selecting a pelvic anatomy template that aligns with your intended visual and didactic objectives. This may involve utilizing a standardized, publicly available anatomical reference or, alternatively, hiring a medical illustrator to develop a customized version tailored to your specific educational or clinical focus. Including the ureters and nerves in the template fosters the radiologist’s credibility.
3	**Start illustrating the findings**. Begin by illustrating the simpler lesions first. Small lesions involving the uterosacral ligaments, or endometriomas, require minimal drawing skills. As you become more familiar with translating the imaging findings onto paper or computer, you will gradually feel more confident in depicting the involvement of more complex structures, such as the parametrium and ureter.
4	**Avoid perfectionism**. At the beginning, you can struggle to accurately illustrate the depth of the lesion or specific anatomical details, such as nerve invasion. If you are not comfortable representing your drawings using a laparoscopic view template, you may incorporate a sagittal view to depict the same findings from a different perspective. Three-dimensional models can also help meet the expectations of radiologists who prefer more literal and detailed visual representations.
5	**Use tools to optimize your workflow**. Look for helpful tools like tablets, drawing apps, and custom brushes. Numerous options and tutorials are available online to help you get started.
6	**Collaborate with colleagues for feedback**. Share your drawings with clinical colleagues and surgeons to receive constructive feedback and improve your illustrations over time.

## Conclusion

Imaging is considered a revolution in the clinical management of endometriosis. Medical illustrations as complements to radiology reports enhance the communication of findings to surgeons and patients, bringing also benefits to the radiologists themselves, being therefore recommended by the relevant radiological society consensus on endometriosis.

## References

[1] Thomassin-Naggara I, Monroc M, Chauveau B et al (2023) Multicenter external validation of the deep pelvic endometriosis index magnetic resonance imaging score. JAMA Netw Open 6:e2311686 37140921 10.1001/jamanetworkopen.2023.11686 PMC10160872

[2] Chamié LP (2020) Ultrasound evaluation of deeply infiltrative endometriosis: technique and interpretation. Abdom Radiol (NY) 45:1648–1658 31740997 10.1007/s00261-019-02322-7

[3] Quesada J, Härmä K, Reid S et al (2023) Endometriosis: a multimodal imaging review. Eur J Radiol 158:110610 36502625 10.1016/j.ejrad.2022.110610

[4] Choi S, Roviglione G, Chou D, D’Ancona G, Ceccaroni M (2024) Nerve-sparing surgery in deep endometriosis: has its time come? Best Pract Res Clin Obstet Gynaecol 96:102506 38981835 10.1016/j.bpobgyn.2024.102506

[5] Barbisan CC, Andres MP, Torres LR et al (2021) Structured MRI reporting increases completeness of radiological reports and requesting physicians’ satisfaction in the diagnostic workup for pelvic endometriosis. Abdom Radiol (NY) 46:3342–3353 33625575 10.1007/s00261-021-02966-4

[6] Becker CM, Bokor A, Heikinheimo O et al (2022) ESHRE guideline: endometriosis. Hum Reprod Open 2022:hoac009 35350465 10.1093/hropen/hoac009 PMC8951218

[7] Borghese G, Coppola F, Raimondo D et al (2022) 3D patient-specific virtual models for presurgical planning in patients with recto-sigmoid endometriosis nodules: a pilot study. Medicina (Kaunas) 58:86 10.3390/medicina58010086 PMC8777715 35056394

[8] Thomassin-Naggara I, Dolciami M, Chamie LP et al (2025) ESUR consensus MRI for endometriosis: indications, reporting, and classifications. Eur Radiol 35:7260–7268 10.1007/s00330-025-11579-0 PMC12559033 40425757

[9] Expert Panel on GYN and OB Imaging, Feldman MK, Wasnik AP et al (2024) ACR Appropriateness Criteria® endometriosis. J Am Coll Radiol 21:S384–S395 39488350 10.1016/j.jacr.2024.08.017

[10] Mattos LA, Goncalves MO, Andres MP et al (2019) Structured ultrasound and magnetic resonance imaging reports for patients with suspected endometriosis: guide for imagers and clinicians. J Minim Invasive Gynecol 26:1016–1025 30849475 10.1016/j.jmig.2019.02.017

[11] Shinagare AB, Lacson R, Boland GW et al (2019) Radiologist preferences, agreement, and variability in phrases used to convey diagnostic certainty in radiology reports. J Am Coll Radiol 16:458–464 30584042 10.1016/j.jacr.2018.09.052

[12] Jha P, Sakala M, Chamie LP et al (2020) Endometriosis MRI lexicon: consensus statement from the Society of Abdominal Radiology endometriosis disease-focused panel. Abdom Radiol (NY) 45:1552–1568 31728612 10.1007/s00261-019-02291-x

[13] Keckstein J, Saridogan E, Ulrich UA et al (2021) The #Enzian classification: a comprehensive non-invasive and surgical description system for endometriosis. Acta Obstet Gynecol Scand 100:1165–1175 33483970 10.1111/aogs.14099

[14] Maciel C, Ferreira H, Djokovic D et al (2023) MRI of endometriosis in correlation with the #Enzian classification: applicability and structured report. Insights Imaging 14:120 37405519 10.1186/s13244-023-01466-x PMC10323073

[15] Abrao MS, Andres MP, Gingold JA et al (2023) Preoperative ultrasound scoring of endometriosis by AAGL 2021 endometriosis classification is concordant with laparoscopic surgical findings and distinguishes early from advanced stages. J Minim Invasive Gynecol 30:363–373 36403696 10.1016/j.jmig.2022.11.003

[16] Rousset P, Buisson G, Lega J-C et al (2021) Rectal endometriosis: predictive MRI signs for segmental bowel resection. Eur Radiol 31:884–894 32851441 10.1007/s00330-020-07170-4

[17] VanBuren W, Feldman M, Shenoy-Bhangle AS et al (2024) Radiology state-of-the-art review: endometriosis imaging interpretation and reporting. Radiology 312:e233482 39287524 10.1148/radiol.233482

[18] Abrao MS, Andres MP, Miller CE et al (2021) AAGL 2021 endometriosis classification: an anatomy-based surgical complexity score. J Minim Invasive Gynecol 28:1941–1950.e1 34583009 10.1016/j.jmig.2021.09.709

[19] Rockall AG, Justich C, Helbich T, Vilgrain V (2022) Patient communication in radiology: moving up the agenda. Eur J Radiol 155:110464 36038410 10.1016/j.ejrad.2022.110464

[20] Kearns C (2019) Is drawing a valuable skill in surgical practice? 100 surgeons weigh in. J Vis Commun Med 42:4–14 10.1080/17453054.2018.1558996 30773071

[21] Gutzeit A, Heiland R, Sudarski S et al (2019) Direct communication between radiologists and patients following imaging examinations. Should radiologists rethink their patient care? Eur Radiol 29:224–231 29943178 10.1007/s00330-018-5503-2

[22] Lauar MCV, Oliveira BC, De Nicola ALA, Chamié LP (2026) Empathetic radiologic approaches in the era of AI: how can we improve the journey of patients with endometriosis? Abdom Radiol (NY) 51:408–416 10.1007/s00261-025-05052-1 40493178

[23] Kovach N, Dix S, Brand G et al (2023) Impact of art and reflective practice on medical education in the emergency department. Emerg Med Australas 35:450–455 36535302 10.1111/1742-6723.14147

[24] Shaffer K, Spittler N (2022) Art and drawing in radiology education. In: Catanzano T (ed) Image-based teaching: techniques, tips and tricks. Springer, pp 87–99

[25] Tjasink M, Keiller E, Stephens M, Carr CE, Priebe S (2023) Art therapy-based interventions to address burnout and psychosocial distress in healthcare workers—a systematic review. BMC Health Serv Res 23:1059 37794353 10.1186/s12913-023-09958-8 PMC10552408

[26] McCarty JL, Gołofit P, Tigges S, Skalski M (2018) Digital medical illustration for the radiologist. Radiographics 38:1145–1157 29856683 10.1148/rg.2018170088

